# Outcomes of Cataract Surgery in Urban and Rural Population in the South Indian State of Andhra Pradesh: Rapid Assessment of Visual Impairment (RAVI) Project

**DOI:** 10.1371/journal.pone.0167708

**Published:** 2016-12-05

**Authors:** Srinivas Marmamula, Rohit C. Khanna, Konegari Shekhar, Gullapalli N. Rao

**Affiliations:** 1 Allen Foster Community Eye Health Research Centre, Gullapalli Pratibha Rao International Centre for Advancement of Rural Eye care, L V Prasad Eye Institute, Hyderabad, India; 2 Brien Holden Institute of Optometry and Vision Science, L V Prasad Eye Institute, Hyderabad, India; 3 Wellcome Trust / Department of Biotechnology India Alliance Research Fellow, L V Prasad Eye Institute, Hyderabad, India; Swinburne University of Technology, AUSTRALIA

## Abstract

**Objective:**

To assess the visual outcomes after cataract surgery among urban and rural population aged ≥40 years in the South India state of Andhra Pradesh.

**Methods:**

A population based cross-sectional study was conducted in which 7800 subjects were sampled from two rural and one urban location. Visual Acuity was assessed and eye examination were performed by trained personnel. A questionnaire was used to collect personal and demographic information, and history of cataract surgery. Blindness and moderate Visual Impairment (MVI) was defined as presenting VA <6/60 and <6/18 to 6/60 in the better eye respectively.

**Results:**

In total, 7378 (94.6%) were examined. Of these, 1228 eyes of 870 individuals were operated for cataract. The mean age of operated subjects was 63.7 years (SD: 10.7 years). Overall, 56.3% of those operated were women, 76% were illiterate and 42% of them were using spectacles after cataract surgery. Even after surgery, 12.2% of the operated eyes had MVI and blindness was seen in 14.7% of the eyes. A significantly higher proportion of subjects in urban area had good outcome as compared to those in the rural area (p = 0.01). Uncorrected refractive error (58.7%) was the leading cause of MVI, and posterior segment disease (34.3%) was the leading cause of blindness. On applying multiple logistic regression, risk factors for poor outcomes were age ≥ 70 years (OR: 1.9, 95% CI: 1.3–2.8), rural residence (OR: 1.3, 95% CI:1.0–1.8) and presence of aphakia (OR: 8.9, 95% CI: 5.7–13.8).

**Conclusions:**

Post cataract surgery, refractive errors remain an important correctable cause of MVI, in the south Indian state of Andhra Pradesh. The correction of refractive errors is required to provide good visual recovery and achieve the benefit of cataract surgery.

## Introduction

Cataract remains the leading cause of visual impairment (VI) worldwide though proportions vary across regions. [1,2] Cataract surgery is one of the most commonly performed ophthalmic surgical procedures, globally. Recent reviews concluded an improved quality of life after cataract surgery. [3,4] Recent decades have witnessed technological advances in cataract surgery and transition from intra capsular cataract extraction with aphakic spectacle correction to phacoemulsification and small incision cataract surgery with intraocular lens (IOL) implantation, and more recently, femtosecond laser assisted cataract surgery. However, despite all these, globally, in most developing countries, there are issues with outcomes of cataract surgeries with poor outcomes ranging from as low as 11.4% to as high as 44%. [5–20]. Most of these study setting was rural [7–9, 12, 13, 15,19] or mix of urban and rural population [10, 11, 14, 16–18, 20] and very few were urban. [5, 6] Risk factors identified in some of these studies included increasing age [8, 11] female gender [5, 10] having no education [11, 13, 14] rural residence [10, 11, 13 14], operated in government sector [11, 13] having free surgery [8, 13] and presence of aphakia. [5, 13, 14]

In India the ‘camp based surgeries’ gave way to ‘hospital based surgeries’ which resulted in better outcomes after cataract surgery, over time. [8,10] In India over 6.3 million cataract surgeries were performed during 2013–2014. [21] Being the most commonly performed surgical procedure that impacts blindness prevention strategies; several researchers have highlighted the importance of monitoring cataract outcomes. [22,23] India, owing to the large size of the country, with huge regional variations in terms of coverage and outcomes, [5,8,10,13,14,24,25] regional surveys are required for local planning of eye care services. [22]

In the year 2011–12, we undertook large population based cross sectional studies using rapid assessment (RA) methodology in one urban (Vijayawada in Krishna district) and two rural locations (Khammam and Warangal district), in the south Indian state of Andhra Pradesh, among those aged 40 years and older. [26] In our earlier papers, we reported on the prevalence and causes of visual impairment, spectacles use and barriers for the uptake of eye care services. [26–28] The overall age and gender adjusted prevalence of visual impairment (VI) in this study population was 14.3%. [26] In this paper we report visual outcomes, causes of VI and risk factors for poor outcome following cataract surgery in this population.

## Materials and Methods

The study protocol was reviewed and approved by Institutional Review board (IRB)(Scientific and Ethics committee) of Hyderabad Eye Research Foundation, L V Prasad Eye Institute, Hyderabad, India. This study adhered to the tenets of the Declaration of Helsinki. The head of the village was explained about the details of the study objectives and procedures by the visiting teams, and consent was obtained to carry out the survey. At household level, the study procedures were explained to each individual and a verbal consent was obtained in presence of other family members and another individual who does not belong to same family, usually a neighbour. Each individual was free to decide on participation in the study. As the study used a simple and non-invasive eye screening protocol, IRB granted permission for verbal consent. The studies were carried out in phases during year 2011 and 2012. The written consent was not obtained as the community where the study was conducted has lower levels of literacy and participants are reluctant to give their thumb impression on the consent form. As this might lead to poor response, IRB was had given approval for verbal consent. [26] In some villages, the verbal consent process in documented in photographs.

The detailed methodology, including sampling procedures has been described elsewhere. [26]

As this study was a part of much larger study that was designed to assess the prevalence of blindness, sample size was not specifically calculated for this study. [26] The sample size was calculated with an expected prevalence of blindness of 6%, precision 20% with 95% error bound, and 10% non-response rate. The sample size is calculated to be approximately 2600 from each of the three study areas and the total sample size was 7800. This sample size is adequate for assessing the visual outcomes after cataract surgery. [26]

A multi-stage sampling procedure was used to select the study sample. [26] In total, 52 clusters are randomly selected from each of the three study districts. In each cluster, the area was demarcated, mapped and segmented in such a way that each segment contained the required number of households to provide at least 50 individuals aged 40 years and older. [26] One of the segments was randomly selected for the study. Villages and municipals wards were used as clusters in rural and urban area respectively. All the individuals in each of the selected household fulfilling the age criteria were listed and all those available were examined. [26] At least two attempts were made to examine those who were not available at the first visit. Those who were still not available after repeated attempts were considered as not available and were not substituted so that bias in recruitment could be minimized. [26]

In brief, one of the three teams, each of which comprised of a vision technician and a community eye health worker, visited the selected households and conducted eye examinations on all the eligible participants. Information on demographics, which include age, gender and education, was collected. Those who have undergone at least primary education were classified as ‘educated.’ Distance visual acuity (VA) was measured using a Snellen chart with tumbling E optotypes at a distance of 6 meters. Presenting and pinhole VA were assessed and for those wearing glasses, additionally unaided VA was assessed. Spectacle use was defined as those who were using spectacles at the time of examination. Torchlight examination and distance direct ophthalmoscopy were performed to assess the lens status in each eye and it was marked as ‘Normal lens’, ‘Cataract’, ‘Pseudophakia’ or ‘Aphakia’. If lens could not be examined, it was marked as ‘No view of lens’ and the reason was documented.

Questions were asked on utilization of eye care services in the past, including cataract surgery and intraocular lens implantation. If a subject reported past cataract surgery, then the place of surgery (government hospital, private hospital, Non-government hospital, in ‘makeshift’ eye camp), cost of surgery (free or paid) and duration of time in years since the surgery were asked. Though makeshift surgical eye camps are no more conducted in India, it was a practice that was widely prevalent more than a decade ago. If the subjects had previous consultation, the reports are reviewed, if available for any further information on ophthalmic consultation. Definitions of cataract and refractive error have been described elsewhere. [26] Cataract was defined as opacity of crystalline lens obscuring the red reflex partially or completely on distant direct ophthalmoscopy and causing visual impairment. Refractive error was deemed to be present if presenting distance VA was worse than 6/18 and improving to 6/18 or better with pinhole.

If there were no media opacities and vision did not improve with pinhole, the cause was attributed to posterior segment pathology. Visual outcome was defined as described by World Health Organization (WHO). [29] Poor outcome after cataract surgery is defined as presenting VA worse than 6/18 in the operated eye. This is based on the surgical history given by the participant and the clinical examination. It was categorised as ‘0’ for good outcome i.e. presenting visual acuity in the operated eye as 6/18 or better and ‘1’ for poor outcome i.e. presenting visual acuity worse than 6/18 in operated eye. Risk factors included age categories (less than 60 years; 60–69 years and more than or equal to 70 years), gender (male versus female), education (any education versus no education), area (urban versus rural), lens status (aphakia versus pseudophakia), duration of time in years since the surgery (less than or equal to 5 years and more than 5 years) place of surgery (government hospital, private hospital, Non-government hospital, in ‘makeshift’ eye camp) and cost of surgery (free or paid).

### Statistical analyses

The data analyses were conducted using Stata 12. [30] The point prevalence of poor outcome estimates were calculated and presented with 95% confidence intervals. The chi-square test was used to test the association between the categorical variables such as poor outcome with age groups, gender and other categorical variables. Univariable and multivariable analysis for risk factor for those having VA less than 6/18 in operated eye was done using generalized estimating equation (GEE) along with robust variance estimation to account for correlation between the two eyes of an individual. The fitness of the regression model was assessed using Hosmer-Lemeshow test for goodness of fit. The adjusted odds ratios (OR) with 95% CI are reported.

## Results

### Baseline characteristics

In total, 7800 subjects aged 40 years and older were enumerated of whom 7378 (94.6%) were examined. The demographic profile of the study participants is published. [26] Of these, 1228 eyes of 870 individuals were operated for cataract. The prevalence of cataract surgery was 11.8% (95% CI: 11.0–12.5) and bilateral cataract surgery was 4.6% (95% CI:4.4–5.3).

Table 1 shows the demographic details of the operated subjects stratified by area of residence (urban versus rural). The mean age of operated subjects was 63.7 years (SD:10.7 years). In total, 56.3% of those operated were women, 76% were illiterate, 42% of them were using spectacles after cataract surgery and 58.9% were cases of unilateral cataract surgery. While those operated upon in urban and rural areas are similar in terms of age (p = 0.33), cataract surgery was more common among women in urban area compared to rural (p = 0.04) and those operated upon in rural area were more likely to be illiterate than those in urban areas (p = 0.04). Bilateral cataract surgery was higher in urban area than in rural areas (p<0.01).

**Table 1:** Characteristics of the sample (n = 870).

	Urban and Rural Combined		Urban		Rural		P Value*
	n	%	n	%	n	%	
**Age group**							0.33
< 60	243	27.9	75	26.6	168	28.6	
60–69	329	37.8	107	37.9	222	37.8	
≥70	298	34.3	100	35.5	198	33.7	
**Gender**							0.04
Male	380	43.7	109	38.7	271	46.1	
Female	490	56.3	173	61.3	317	53.9	
**Education**							0.04
No Education	661	76.0	202	71.6	459	78.1	
Any education	209	24.0	80	28.4	129	21.9	
**Spectacle use**							0.69
Yes	365	42.0	121	42.9	244	41.5	
No	505	58.0	161	57.1	344	58.5	
**Cataract Surgery**							<0.01
Unilateral	512	58.9	136	48.2	376	63.9	
Bilateral	358	41.1	146	51.8	212	36.1	
	**870**	100.0	**282**	100.0	**588**	100.0	

*: P-value is comparing urban versus rural

Table 2 shows the ocular characteristics of operated eyes stratified by urban and rural population. IOL implantation was seen in 86.2% of eyes. Nearly two third of the surgeries were performed in the last five years. In total, 38.8% of surgeries were conducted in government hospitals and 30.5% in hospitals managed by non-government organization. Overall 71.3% of the surgeries were done free of cost. Good outcomes were seen in 73% of the operated eyes. As compared to rural areas, those in urban areas had higher proportion of surgeries done in private clinic (44.2% versus 20.9%; p<0.01) and had lower prevalence of free surgeries done (54.4% versus 80.4%; p<0.01). A higher proportion of subjects in urban area had good outcome as compared to rural area (77.6% versus 70.6%; p = 0.01). The visual outcome was also better among the eyes with pseudophakia compared to aphakia (Table 3).

**Table 2:** Characteristics of the Operated eyes (n = 1228).

	Urban and Rural Combined		Urban		Rural		
	n	%	n	%	n	%	p
**Lens Status**							
Aphakia	145	11.8	57	13.3	88	11.0	0.23
Pseudophakia	1058	86.2	368	86.0	690	86.3	0.90
No view of lens	25	2.0	3	0.7	22	2.8	0.02
**Years since surgery**							
< 5 years	798	65.0	286	66.8	512	64.0	0.32
6–10 years	286	23.3	97	22.7	189	23.6	0.70
> 10 years	144	11.7	45	10.5	99	12.4	0.33
**Place of surgery**							
Eye camp	21	1.7	8	1.9	13	1.6	0.75
Government hospital	476	38.8	122	28.5	354	44.3	<0.01
Non-Government Hospital	375	30.5	109	25.5	266	33.3	0.01
Private clinic	356	29.0	189	44.2	167	20.9	<0.01
**Cost of surgery**							
Free surgery	876	71.3	233	54.4	643	80.4	<0.01
Paid surgery	352	28.7	195	45.6	157	19.6	
**Visual Impairment**							
≥ 6/18	897	73.0	332	77.6	565	70.6	0.01
<6/18–6/60	150	12.2	37	8.6	113	14.1	0.01
< 6/60	181	14.7	59	13.8	122	15.3	0.49
	**1228**	**100**	**428**	**100**	**800**	**100**	

* p value comparing rural and urban sample

**Table 3:** Categories of visual impairment stratified by Aphakia and pseudophakia.

	Aphakia	Pseudophakia	Total	p
Presenting visual acuity	n (%)	n (%)	n (%)	
≥ 6/18	39 (26.9)	858 (81.1)	897 (74.6)	<0.01
< 6/18–6/60	31 (21.4)	119 (11.2)	150 (12.5)	<0.01
< 6/60	75 (51.7)	81 (7.7)	156 (13.0)	<0.01
	145 (100)	1058 (100)	1203^†^(100)	

^†^ Excluded 25 eyes where lens could not be examined either due to absent globe or phthisis bulbi

Table 4 shows the causes of VI in urban and rural areas. Uncorrected refractive errors accounted for 28.7% of the VI followed by posterior segment diseases which accounted for 26.9% and surgery related complications accounted for 21.1%. Following refractive error, posterior segment disorder predominated in urban areas, surgery related complications predominated in rural areas, as cause for MVI. Similarly, posterior segment disease, uncorrected aphakia and surgery related complications were the leading causes of blindness after cataract surgery in operated eyes in both urban and rural areas.

**Table 4:** Causes of visual impairment in operated eyes.

	Urban and Rural combined	Urban		Rural	
	All visual impairment	<6/18–6/60	<6/60	<6/18–6/60	<6/60
	n = 331	n = 37	n = 59	n = 113	n = 122
Refractive Error	95 (28.7)	17 (45.9)	0 (0.0)	71 (62.8)	7 (5.7)
Posterior segment disorder	89 (26.9)	13 (35.1)	22 (37.3)	14 (12.4)	40 (32.8)
Uncorrected Aphakia	61 (18.4)	2 (5.4)	26 (44.1)	7 (6.2)	26 (21.3)
Surgery related complications	70 (21.1)	4 (10.8)	9 (15.3)	21 (18.6)	36 (29.5)
Corneal scar	15 (4.5)	1 (2.7)	2 (3.4)	0 (0.0)	12 (9.8)
Others	1 (0.3)	0 (0.0)	0 (0.0)	0 (0.0)	1 (0.8)
	331 (100)	37 (100)	59 (100)	113 (100)	122 (100)

Table 5 shows the risk factors for poor outcome (<6/18) after cataract surgery. Visual outcomes were poor among those aged ≥ 70 years (OR: 1.9, 95% CI: 1.3–2.8); among rural residents (OR: 1.3, 95% CI:1.0–1.8). and in those with aphakia (OR: 8.9, 95% CI: 5.7–13.8). The odds of having poor outcome among those operated upon more than 5 years ago were of borderline significance (OR: 1.3; 95% CI: 0.9–1.8). The visual outcomes were not associated with gender, education, place of surgery and whether the participant paid up or was operated upon for free.

**Table 5:** Logistic regression analysis showing the risk factors for poor visual outcomes after cataract surgery (n = 1203 eyes).

	Unadjusted Odds ratio	95% CI	Adjusted Odds Ratio	95% CI
**Age group (years)**				
< 60	1.0		1.0	
60–69	1.3	0.9–1.9	1.0	0.7–1.5
≥70	2.8	2.0–4.0	1.9	1.3–2.8
**Gender**				
Male			1.0	
Female	1.0	0.8–1.3	0.9	0.6–1.2
**Education**				
Any education			1.0	
No education	1.9	1.4–2.6	1.6	1.0–2.4
**Area**				
Urban			1.0	
Rural	1.4	1.1–2.0	1.3	1.0–1.8
**Lens Status**				
Pseudophakia			1.0	
Aphakia	11.7	7.8–17.4	8.9	5.7–13.8
**Duration since surgery**				
< = 5 years				
> 5 years	2.6	2.0–3.3	1.3	0.9–1.8
**Place of surgery**				
Private clinic			1	
NGO Hospital	1.4	1.0–2.0	1.2	0.6–2.1
Govt hospital	2.6	1.9–3.6	1.7	0.9–3.3
Make-shift eye camp	6.2	2.5–15.3	0.8	0.2–2.2
**Cost of surgery**				
Paid			1.0	
Free	2.1	1.6–2.9	1.2	0.6–2.2

## Discussion

We reported population based visual outcomes after cataract surgery in the south Indian state of formerly undivided Andhra Pradesh, India and reported the urban and rural differences. Though different outcome based studies in India had both urban and rural population, [10,11] only Chennai Glaucoma Study (CGS) [14] reported the urban and rural differences in outcomes. The overall prevalence was 11.8% and was comparable with some of the studies done in India [5,8,10,13,14,25] but higher than those reported from neighboring countries such as Nepal, Bangladesh and China. [6,7,12,31] Unlike, CGS, there was no difference in the prevalence of cataract surgery in urban and rural areas (11.9% versus 11.5%; p = 0.56), suggesting an increase in uptake of surgeries in rural areas too.

Overall, 56.3% of surgeries were done in female patients and similar trends were seen across other studies in India. [8,10,11,13,14,25] It’s known that females live longer and show higher prevalence of cataract surgery, which could be one of the reasons for more number of surgeries in female patients. The percentage of surgeries in females in the urban area was higher than in rural areas and this could be likely due to increased awareness and education in females in urban areas as compared to rural areas. This could also explain the reason for the higher prevalence of bilateral cataract surgeries as well as surgeries on literates in urban areas as compared to rural areas.

Overall 86.2% of surgeries were with IOL and unlike CGS, [14] there was no difference between urban and rural areas. Our study was almost a decade later than CGS and nearly two-third of surgeries in our cohort were done in the last 5 years. This is a welcome trend suggesting higher IOL implantation in rural areas too. Most of the surgeries in urban areas were done in private clinics and in rural areas by government and non-governmental organization (NGO) hospitals. This is due to the fact that most of the private clinics are located in urban areas and government and NGO hospitals operate upon most patients from rural areas through their outreach programs.

Overall visual acuity of more than 6/18 was seen in more than 70% of the participants. This was much higher than those reported from other studies in India and other developing countries, [5–7,10–14,25,31,32] however this was similar to one of the recent studies. [8] It could be due to the fact that, as compared to other studies, the percentage of aphakia was lower in our study. Outcomes were also good among pseudophakic participants as compared to aphakic participants due to use of IOLs. Apart from that, with time, more and more sutureless surgeries (manual small incision cataract surgery and phacoemulsification) are being performed across the country, thus causing less astigmatism and better uncorrected visual acuity.

Like other studies, [5,8,10,13,14,24,25] increasing age was a risk factor for poor outcome and it is likely that with increasing age, there are other co-existing morbidities, which could affect outcomes. Similarly, those in rural areas were also having poor outcomes and it could be due to the fact that most of these surgeries were done in government and NGO hospitals, including free of cost surgeries. Most of these surgeries in rural areas are done through outreach programs as part of the National Program for Control of Blindness (NPCB) activity where they are transported to the base hospital for surgeries and given one-time free glasses six weeks after cataract surgery. It is seen that, if the glasses are broken or lost, many of these patients do not get a replacement of a new pair of glasses, and manage with the existing vision, thus affecting outcomes. Outcomes were also better with the use of spectacles as well as in pseudophakia. The major cause of MVI in urban and rural areas was refractive error followed by posterior segment disorder in urban areas and surgery related complications in rural areas.

The major cause of blindness in both urban and rural areas was posterior segment disorder uncorrected aphakia and surgery related complications. This was similar to other studies in India too. [5,10,13,14,24,25,32] One of the major strengths of this study was large sample as well as urban and rural mix, giving an opportunity to look at the differences. Apart from this, the use of rapid assessment methods allows comparison with other studies using similar methodology. One of the major limitation of the study was use of pinhole visual acuity as a surrogate for best-corrected visual acuity. To overcome this, we have used the presenting visual acuity for our analysis.

Apart from this, another limitation was attributing posterior segment disorder as the cause of VI if the vision did not improve with pinhole and the media was clear. This is likely to lead to an overestimate of posterior segment disorder as the cause of VI. Apart from this other limitation include non-availability of pre-operative, intra-operative as well as post-operative surgical records of these participants, limiting the ability to pin point the exact cause of uncorrected refractive error.

In summary, the study showed a trend in improvement of outcomes in population as compared to previous study. However, the outcomes are still below the accepted WHO norms. [29] One of the major reasons was lack of use of spectacles by more than 50% of the participants in the post-operative period. Hence, emphasis should also be paid to ensuring regular use of spectacles in the post-operative period as well as later.
